# Computed tomography-measured body composition and survival in rectal cancer patients: a Swedish cohort study

**DOI:** 10.1186/s40170-022-00297-6

**Published:** 2022-11-23

**Authors:** Angeliki Kotti, Annica Holmqvist, Mischa Woisetschläger, Xiao-Feng Sun

**Affiliations:** 1 Department of Radiology in Linköping, and Department of Health, Medicine and Caring Sciences, Linköping University, SE-581 85 Linköping, Sweden; 2 Department of Biomedical and Clinical Sciences, Linköping University, SE-581 83 Linköping, Sweden; 3 Department of Oncology, and Department of Biomedical and Clinical Sciences, Linköping University, SE-581 85 Linköping, Sweden; 4 Centre for Medical Image Science and Visualization (CMIV), Linköping University, SE-581 85 Linköping, Sweden

**Keywords:** Rectal cancer, Body composition, Skeletal muscle, Obesity, Radiotherapy, Survival

## Abstract

**Background:**

The association between body composition and survival in rectal cancer patients is still unclear. Therefore, we aimed to evaluate the impact of computed tomography (CT)-measured body composition on survival in rectal cancer patients, stratifying our analyses by sex, tumour location, tumour stage and radiotherapy.

**Methods:**

This retrospective cohort study included 173 patients with rectal adenocarcinoma. CT colonography scans at the time of diagnosis were used to assess the skeletal muscle index (SMI) and the visceral adipose tissue area (VAT). The patients were divided into a low or high SMI group and a low or high VAT group according to previously defined cutoff values. Endpoints included cancer-specific survival (CSS) and overall survival (OS).

**Results:**

In all patients, low SMI was associated with worse CSS (HR, 2.63; 95% CI, 1.35–5.12; *P* = 0.004) and OS (HR, 3.57; 95% CI, 2.01–6.34; *P* < 0.001) compared to high SMI. The differences remained significant after adjusting for potential confounders (CSS: adjusted HR, 2.28; 95% CI, 1.13–4.58; *P* = 0.021; OS: adjusted HR, 3.17; 95% CI, 1.73–5.82; *P* < 0.001). Low SMI was still related to a poor prognosis after stratifying by sex, tumour location, stage and radiotherapy (*P* < 0.05). High VAT was associated with better CSS (HR, 0.31; 95% CI, 0.11–0.84; *P* = 0.022) and OS (HR, 0.40; 95% CI, 0.17–0.97; *P* = 0.044) compared to low VAT among men with rectal cancer ≤ 10 cm from the anal verge. High VAT was associated with worse CSS (HR, 4.15; 95% CI, 1.10–15.66; *P* = 0.036) in women with rectal cancer ≤ 10 cm from the anal verge.

**Conclusions:**

Low SMI was associated with worse survival. High VAT predicted better survival in men but worse survival in women. The results suggest that CT-measured body composition is a useful tool for evaluating the prognosis of rectal cancer patients and demonstrate the need to include the sex and the tumour location in the analyses.

**Supplementary Information:**

The online version contains supplementary material available at 10.1186/s40170-022-00297-6.

## Background

In recent years, body composition parameters have been suggested as prognostic factors in patients with different malignancies [1–3]. Previous studies have found that sarcopenia, defined as reduced skeletal muscle mass, and visceral obesity were related to survival in cancer patients [4, 5]. For the assessment of body composition, computed tomography (CT) scans acquired during routine clinical care have been used in recent years, as this method allows the accurate quantification of both the skeletal muscle and adipose tissue [6, 7].

In rectal cancer, the evidence for the effects of body composition on clinical outcomes is inconclusive. Some studies show a relationship of reduced muscle mass and visceral obesity with poor prognosis [8–10], while others report no such association [11]. A recent meta-analysis on the prognostic value of sarcopenia in colorectal cancer showed no difference in survival when the subgroup of rectal cancer patients was analysed separately [12].

As body composition differs between men and women [13], studies on body composition and clinical outcomes should not only use sex-specific cutoff values but even analyse the data in men and women separately [14]. Moreover, since an association between the distance of cancer from the anal verge and the outcomes has been reported [15], the tumour location seems to be an important factor in rectal cancer research. Few studies considered the tumour location when analysing the role of body composition [16, 17].

Another important area in the research on body composition is the development of pharmacological treatments to improve patients’ muscle mass. To date, no drugs have been approved for the treatment of sarcopenia. Previous studies have reported an association between low skeletal muscle mass and the presence of systemic inflammation [18]. It has been suggested that beta-adrenergic receptor antagonists (beta-blockers), a category of drugs primarily used in cardiovascular disease, can modulate the inflammatory response [19]. Thus, one can hypothesize that beta-blockers might have an impact on skeletal muscle mass.

Taken together, it is interesting to assess the potential prognostic role of body composition parameters in rectal cancer patients, considering both the patient’s sex and the tumour location. Additionally, since radiotherapy (RT) is an established treatment against rectal cancer, an investigation into the role of body composition in patients who underwent RT seems meaningful. Moreover, to our knowledge, no one has studied the association between beta-blocker use and CT-measured body composition in patients with rectal cancer.

The aim of the present study was to evaluate the impact of CT-measured body composition in terms of skeletal muscle mass and visceral adipose tissue on clinical outcomes in patients with rectal cancer, stratifying the analyses by sex, tumour location, tumour stage and preoperative RT. Furthermore, we investigated whether there was any association between the use of beta-blockers and body composition in rectal cancer patients.

## Methods

### Study cohort

**Fig. 1:**
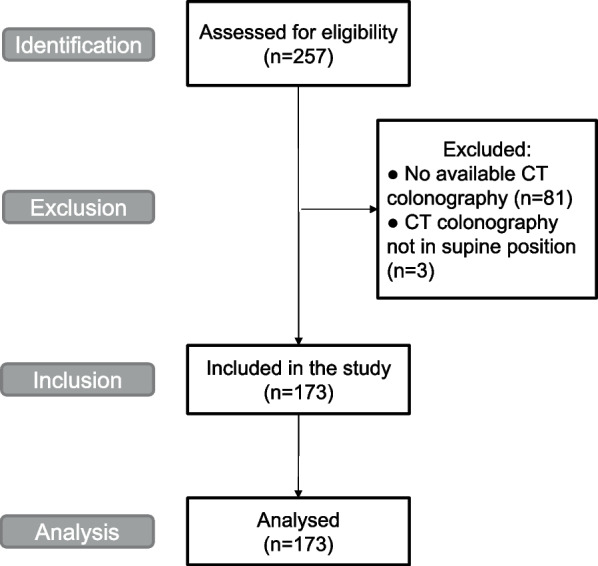
Flow diagram of the patient inclusion process

By using the Swedish Colorectal Cancer Register, we initially identified 257 patients from the region of Ostergotland, in Sweden, who had been diagnosed with rectal adenocarcinoma between 2008 and 2012. By using the Picture Archiving and Communication System (PACS) in the Department of Radiology at Linkoping University Hospital, we identified those patients who underwent a CT colonography in a supine position at the time of diagnosis. Finally, 173 patients were enrolled in the study, and the selection process is summarized in Fig. 1.

The surgical, oncological and radiological records of each patient were reviewed retrospectively, and data including the following was collected: date of diagnosis, sex, age at diagnosis, weight at the time of CT colonography, height within 6 months of the date of CT colonography, tumour location (distance from the anal verge to the lower border of rectal cancer), clinical TNM stage, differentiation, rectal cancer surgery, type of RT, neoadjuvant/adjuvant chemotherapy, history of comorbidities at the time of diagnosis (coronary artery disease, diabetes and/or hypertension), use of any type of beta-blockers at the time of diagnosis and survival. Data on the causes of death was obtained from the Death Register of the Swedish National Board of Health and Welfare.

For the subgroup analyses by tumour location, the patients were divided into two groups: those with cancer ≤ 10 cm from the anal verge and those with cancer > 10 cm [15, 17]. In the subgroup analyses by RT, the patients with stage II and III disease who received preoperative RT were included. In the subgroup analyses of SMI and beta-blockers, the patients were divided into two groups, those who were using beta-blockers at the time of diagnosis and those who were not. The variable of age was used as a dichotomous variable (younger/older) with the mean age of the study population (69 years) as the cutoff value.

### Body composition measurements

**Fig. 2:**
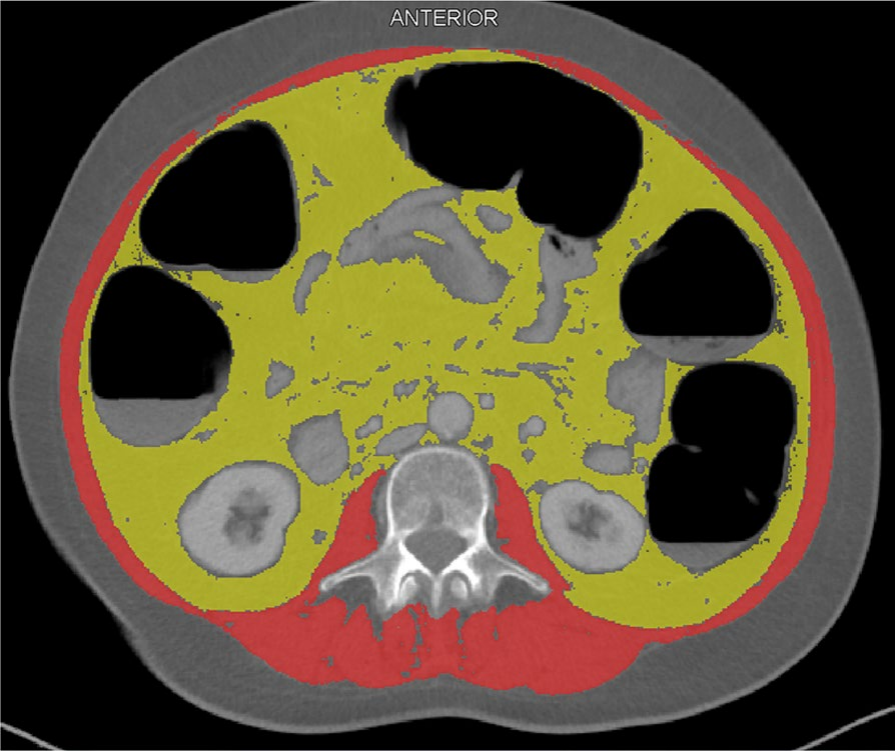
Segmentation of skeletal muscle area (red) and visceral adipose tissue area (yellow) in an axial CT image at the L3 vertebral body using the Slice-O-Matic software

The measurements of body composition were performed using each patient’s CT scan, acquired at the time of rectal cancer diagnosis. None of the patients had received any treatment for rectal cancer before the CT scan. The CT scans were obtained from the Picture Archiving and Communication System (PACS) in the Department of Radiology at the University Hospital of Linköping, Sweden. Of the 173 analysed CT scans, 167 were performed with intravenous contrast in the portal venous phase, while 6 were performed without intravenous contrast. A resident in radiology with 4 years of training (AK) quantified the skeletal muscle area (SMA) and visceral adipose tissue area (VAT), while blinded to clinicopathological data. One 5-mm-thick axial slice made in a supine position was selected at the level of the third lumbar vertebral body (L3) transverse processes. L3 SMA (cm^2^) and VAT (cm^2^) were quantified, using the Slice-O-Matic software (version 5.0, Tomovision, Canada). SMA included erector spinae, psoas, quadratus lumborum, rectus abdominis, transversus abdominis and internal and external oblique (Fig. 2). A Hounsfield unit (HU) threshold of − 29 to + 150 was used to identify SMA, and a HU threshold of − 150 to − 50 was used for VAT. The SMA was then divided by the square of body height and expressed as skeletal muscle index (SMI, cm^2^/m^2^) [6, 20]. BMI was calculated as weight (kg)/height (m^2^).

The study population was divided into a low and a high SMI group using sex-specific cutoff points established in a previous study on a Caucasian population [21]. Low SMI was defined as under 41.6 cm^2^**/**m^2^ for men and under 32 cm^2^/m^2^ for women. For the analysis of VAT, the patients were divided into two groups: a low and a high VAT group. We defined low VAT as under 160 cm^2^ for men and under 80 cm^2^ for women, using the thresholds proposed in previous studies [22, 23]. The cutoff point for BMI was set at 25 kg/m^2^ for both men and women, according to the WHO definition of overweight and obesity.

### Outcomes

The outcomes were cancer-specific survival (CSS) and overall survival (OS) measured from the date of diagnosis to the date of the event or the last follow-up. For the CSS, deaths due to rectal cancer were treated as events, and deaths from other causes were treated as censored observations.

### Statistical analysis

The chi-squared test or the Fisher exact test was used for the comparison of categorical variables. The *t*-test or Mann-Whitney *U*-test was used for continuous variables. Survival curves were calculated according to the Kaplan-Meier method and compared using the log-rank tests. Univariate and multivariate Cox proportional hazard models were used to estimate hazard ratios (HR), adjusted hazard ratios (aHR) and corresponding 95% confidence intervals (CI). The variables included in the multivariate Cox model as potential confounders were defined a priori: (i) for CSS, we adjusted for age, stage and differentiation, and (ii) for OS, we adjusted for age, stage, differentiation and history of coronary artery disease at the time of diagnosis. Coronary artery disease was selected as a potential confounder, since both sarcopenia and obesity are associated with coronary artery disease [24, 25]. The proportional hazard assumption was checked by using statistical tests [26]. Missing data were excluded from the analysis. All statistical analyses were performed using the Statistica software, with *P* < 0.05 considered statistically significant.

## Results

### Baseline characteristics

A total of 173 rectal cancer patients were included in the study. The mean age of the patients at diagnosis was 69 years (range 29–91 years). There were 102 (59%) men and 71 (41%) women. The mean follow-up duration was 46 months (range 2–60 months). In men, 26 rectal cancer deaths (26%) were observed compared to 11 (16%) among women (*P* = 0.097). In men, 35 all-cause deaths (35%) were observed compared to 16 (23%) among women (*P* = 0.229). Thirty-eight per cent (*n* = 39) of all men were using beta-blockers at the time of diagnosis compared to 38% (*n* = 27) of women (*P* = 0.978). In women, the use of beta-blockers was related to older patients (*P* < 0.001), but not to men (*P* = 0.314). Among men or women, no statistically significant association between the use of beta-blockers and BMI (*P* = 0.077; *P* = 0.347, respectively) stage (*P* = 0.135; *P* = 0.392, respectively) or differentiation (*P* = 0.881; *P* = 0.211, respectively) was observed. Other baseline characteristics are presented in Table 1.

**Table 1:** Rectal cancer patients’ characteristics according to sex

	Men, ***n*** (%)	Women, ***n*** (%)
**Age in years, mean [range]**	70 [46–88]	69 [29–91]
**Stage**		
I	16 (18)	13 (16)
II	19 (19)	16 (23)
III	47 (46)	35 (49)
IV	20 (20)	7 (10)
**Differentiation grade**		
Good	9 (9)	6 (8)
Moderate	66 (65)	43 (61)
Poor	27 (26)	22 (31)
**Tumour location**		
Low (≤ 5cm)	39 (38)	22 (31)
Middle (> 5–10 cm)	34 (33)	28 (39)
Upper (> 10–15 cm)	29 (29)	21 (30)
**RT**^**a**^		
Short-course RT^b^	51 (50)	32 (45)
Long-course RT^c^	3 (3)	0
Long-course CRT^d^	11 (11)	15 (21)
Palliative RT	1 (1)	2 (3)
No RT	36 (35)	22 (31)
**Surgery**^**a**^		
Yes	94 (92)	68 (96)
No	8 (8)	3 (4)
**Neoadjuvant chemotherapy**		
Oxaliplatin-based	17 (17)	11 (16)
Non-oxaliplatin-based	2 (2)	1 (1)
No	83 (81)	59 (83)
**Adjuvant chemotherapy**		
Oxaliplatin-based	16 (16)	8 (11)
Non-oxaliplatin-based	12 (12)	7 (10)
No	74 (72)	56 (79)
**History of coronary artery disease**^**e**^		
Yes	10 (10)	6 (8)
No	92 (90)	65 (92)
**History of diabetes**^**e**^		
Yes	15 (15)	9 (13)
No	87 (85)	62 (87)
**History of hypertension**^**e**^		
Yes	40 (39)	27 (38)
No	62 (61)	44 (62)
**Use of beta-blockers**^**e**^		
Yes	39 (38)	27 (38)
No	63 (62)	44 (62)
**Body mass index**		
< 25	42 (41)	33 (47)
≥ 25	54 (53)	35 (49)
Missing data	6 (6)	3 (4)
**SMI cm**^**2**^**/m**^**2**^**, mean (± SD)**	45.8 (± 6.6)	36.2 (± 6.2)
**VAT cm**^**2**^**, mean (± SD)**	166.7 (± 98.3)	83.9 (± 58.9)

*RT* radiotherapy, *CRT* chemoradiotherapy, *SMI* skeletal muscle index, *VAT* visceral adipose tissue area, *SD* standard deviation

^a^For the rectal tumour

^b^25 Gy

^c^50 Gy

^d^44–50.4 Gy

^e^At the time of rectal cancer diagnosis

### SMI in relation to clinical characteristics and survival

Firstly, we assessed SMI in relation to clinical characteristics and survival. One patient was excluded from the SMI analyses due to missing data on height. Of the remaining 172 patients, 43 (25%) had low SMI at the time of diagnosis, and low SMI was associated with older patients (*P* < 0.001). More patients had BMI < 25 in the low SMI group compared to the high SMI group (75% vs. 36%, *P* < 0.001). Besides, there was no relationship between SMI and sex (*P* = 0.655), stage (*P* = 0.98) or differentiation (*P* = 0.380).

**Fig. 3:**
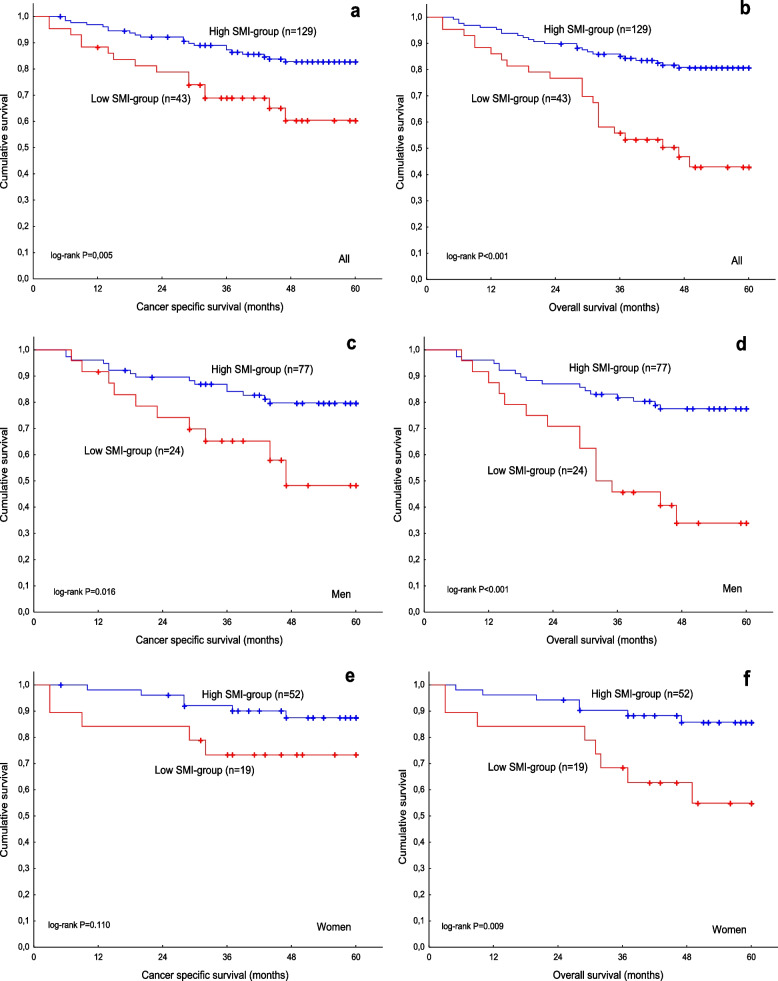
Cancer-specific survival (**a**) and overall survival (**b**) in relation to SMI in all patients. Cancer-specific survival (**c**) and overall survival (**d**) in relation to SMI in men. Cancer-specific survival (**e**) and overall survival (**f**) in relation to SMI in women

Next, we studied the association between SMI and survival. In all patients, low SMI was significantly related to worse CSS and OS (Fig. 3 a, b) compared to high SMI. After adjusting for potential confounders, the difference in CSS and OS remained significant (Table 2). In men, low SMI was related to worse CSS (HR, 2.77; 95% CI, 1.23–6.20; *P* = 0.013; Fig. 3 c) and OS (HR, 3.63; 95% CI, 1.80–7.29; *P* < 0.001; Fig. 3 d). In women, low SMI was associated with worse OS (HR, 3.68; 95% CI, 1.33–10.19; *P* = 0.012; Fig. 3 f), although the significance was lost with CSS (HR, 2.62; 95% CI, 0.78–8.60; *P* = 0.112; Fig. 3 e). There was no statistically significant interaction between SMI and sex for CSS (*P* = 0.983) or OS (*P* = 0.240) in all patients.

**Table 2:** Cancer-specific survival and overall survival for skeletal muscle index in all patients (*n*=172)

	** HR **	**95% CI **	*P*-value	** aHR **	**95% CI **	*P*-value
**Cancer-specific survival**						
High SMI	1.00	Reference		1.00	Reference	
Low SMI	2.63	1.35-5.12	0.004	2.28¹	1.13-4.58	0.021
**Overall survival**						
High SMI	1.00	Reference		1.00	Reference	
Low SMI	3.57	2.01-6.34	<0.001	3.17²	1.73-5.82	<0.001

Abbreviations: HR: hazard ratio; CI: confidence interval; aHR: adjusted hazard ratio ¹ adjusted for age, stage, and differentiation ; ² adjusted for age, stage, differentiation, and history of coronary artery disease

We also studied the association between SMI and survival in the subgroups according to tumour location. In all the patients with cancer ≤ 10 cm from the anal verge (*n* = 122), low SMI was associated with worse CSS (HR, 2.22; 95% CI, 1.08–4.59; *P* = 0.031) and OS (HR, 2.70; 95% CI, 1.41–5.18; *P* = 0.003) compared to high SMI. Among the subgroups of men (*n* = 72) and women (*n* = 50) with cancer ≤ 10 cm, low SMI was related to worse OS (HR, 2.52; 95% CI, 1.09–5.85; *P* = 0.032 and HR, 2.94; 95% CI, 1.03–8.38; *P* = 0.044, respectively) but not to CSS (HR, 2.15; 95% CI, 0.85–5.42; *P* = 0.104 and HR, 2.42; 95% CI, 0.74–7.95; *P* = 0.144, respectively). Among all the patients with cancer > 10 cm from the anal verge (*n* = 50), low SMI was associated with worse OS (HR, 9.25; 95% CI, 2.37–36.17; *P* = 0.001) and tended to be associated with worse CSS (HR, 5.99; 95% CI, 0.97–36.81; *P* = 0.053). Among men with cancer > 10 cm from the anal verge (*n* = 29), low SMI was related to worse CSS (HR, 6.17; 95% CI, 1.01–37.80; *P* = 0.049) and OS (HR, 8.33; 95% CI, 2.06–33.70; *P* = 0.003). We were not able to perform analyses in women with cancer > 10 cm from the anal verge (*n* = 21) due to a few events.

Moreover, we investigated the association between SMI and survival in each tumour stage. In stage II, low SMI was related to worse CSS compared to high SMI (HR, 7.77; 95% CI, 1.02–32.61; *P* = 0.047) but not in stage I, III or IV (Additional file 2: Table S1). The relationship between low SMI and worse OS was statistically significant in stages II (HR, 11.15; 95% CI, 2.33–53.34; *P* = 0.003) and III (HR, 3.33; 95% CI, 1.18–9.38; *P* = 0.022), but not in stage I or IV (Additional file 2: Table S1).

We further investigated the relationship between the use of beta-blockers and SMI in men and women. Among 38 men who used beta-blockers, 11% had low SMI, compared to 32% among non-users (*P* = 0.015). Of 27 women who used beta-blockers, 41% had low SMI compared to 18% among non-users (*P* = 0.037).

### VAT in relation to clinical characteristics and survival

Secondly, we assessed the association between VAT and clinical characteristics as well as survival. Fifty men (49%) had high VAT, compared to 33 women (46%) (*P* = 0.742). The percentage of patients with high BMI (> 25) and high SMI was greater in the high VAT group compared to the low VAT group (82% vs. 28%, *P* < 0.001; 82% vs. 68%, *P* = 0.043, respectively). There was no association between VAT and age (*P* = 0.907), stage (*P* = 0.992) or differentiation (*P* = 0.128).

**Fig. 4:**
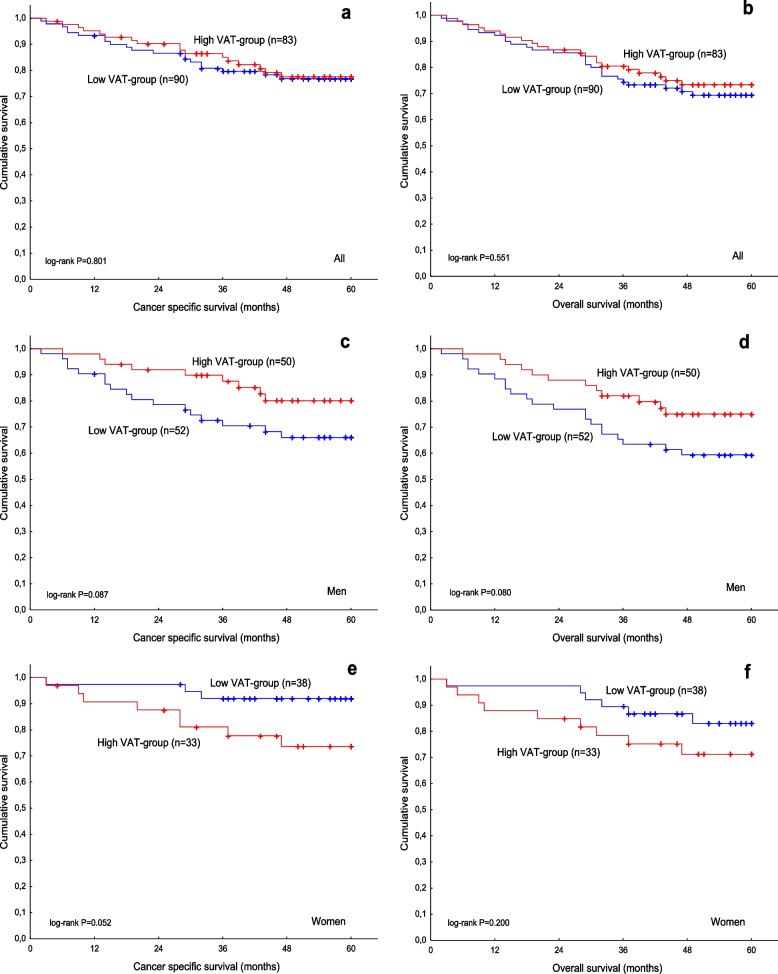
Cancer-specific survival (**a**) and overall survival (**b**) in relation to VAT in all patients. Cancer-specific survival (**c**) and overall survival (**d**) in relation to VAT in men. Cancer-specific survival (**e**) and overall survival (**f**) in relation to VAT in women

In all patients, VAT was not significantly related to either CSS (HR, 0.92; 95% CI, 0.48–1.76; *P* = 0.801; Fig. 4 a) or OS (HR, 0.84; 95% CI, 0.48–1.49; *P* = 0.554; Fig. 4 b). Among men, patients with high VAT tended to have better CSS (HR, 0.50; 95% CI, 0.22–1.13; *P* = 0.095; Fig. 4 c) and OS (HR, 0.54; 95% CI, 0.26–1.09; *P* = 0.086; Fig. 4 d) compared to patients with low VAT. In contrast, among women, there was a tendency for a difference in CSS, with patients with high VAT having worse CSS compared to those with low VAT (HR, 3.46; 95% CI, 0.92–13.04; *P* = 0.067; Fig. 4 e). No such difference was observed for OS in women (HR, 1.94; 95% CI, 0.69–5.47; *P* = 0.207; Fig. 4 f). A statistically significant interaction was found between VAT and sex for CSS (*P =* 0.015) as well as for OS (*P* = 0.043) in all patients.

**Fig. 5:**
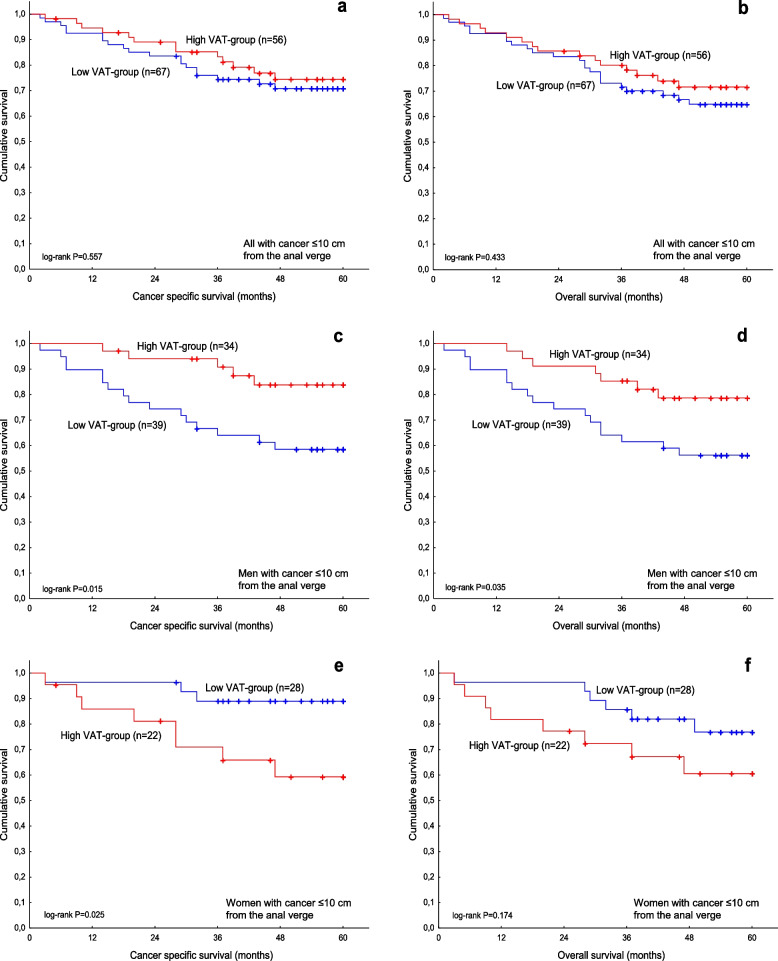
Cancer-specific survival (**a**) and overall survival (**b**) in relation to VAT in all patients with cancer ≤ 10 cm from the anal verge. Cancer-specific survival (**c**) and overall survival (**d**) in relation to VAT in men with cancer ≤ 10 cm from the anal verge. Cancer-specific survival (**e**) and overall survival (**f**) in relation to VAT in women with cancer ≤ 10 cm from the anal verge

We also studied the association between VAT and survival in the subgroups according to tumour location. In all patients with cancer ≤ 10 cm from the anal verge, there was no association of VAT with CSS (HR, 0.81; 95% CI, 0.40–1.64; *P* = 0.560; Fig. 5 a) or OS (HR, 0.77; 95% CI, 0.40–1.48; *P* = 0.437; Fig. 5 b). In men with cancer ≤ 10 cm from the anal verge, high VAT was associated with better CSS (HR, 0.31; 95% CI, 0.11–0.84; *P* = 0.022; Fig. 5 c) and OS (HR, 0.40; 95% CI, 0.17–0.97; *P* = 0.044; Fig. 5 d) compared to low VAT. In women with cancer ≤ 10 cm from the anal verge, high VAT was associated with worse CSS (HR, 4.15; 95% CI, 1.10–15.66; *P* = 0.036; Fig. 5 e) compared to low VAT, while there was no association between VAT and OS (HR, 2.07; 95% CI, 0.72–5.98; *P* = 0.179, Fig. 5 f). There was a statistically significant interaction between VAT and sex for CSS (*P* = 0.002) as well as for OS (*P* = 0.020) in all patients with cancer ≤ 10 cm from the anal verge. In men with cancer > 10 cm from the anal verge, there was no association between VAT and CSS (HR, 3.31; 95% CI, 0.37–29.67; *P* = 0.284) or OS (HR, 1.05; 95% CI, 0.28–3.91; *P* = 0.943). We were not able to perform survival analyses for women with cancer > 10 cm from the anal verge due to a few events.

In addition, we studied the association between VAT and survival according to the tumour stage. In all patients, there was no association between VAT and either CSS or OS when analysed by stage (Additional file 3: Table S2). Men with high VAT had better CSS (HR, 0.26; 95% CI, 0.07–0.94; *P* = 0.040) and OS (HR, 0.27; 95% CI, 0.07–0.96; *P* = 0.044) compared to men with low VAT in stage IV. There were no such differences in CSS or OS among men with stage I, II or III disease (Additional file 3: Table S2). Among women, there were no statistically significant differences in survival when analysed by stage (Additional file 3: Table S2).

We studied the relationship between the use of beta-blockers and VAT in both men and women. Among 39 men who used beta-blockers, 67% had high VAT, compared to 38% among non-users (*P* = 0.005). Of 27 women who used beta-blockers, 56% had high VAT compared to 41% among non-users (*P* = 0.230).

### SMI and VAT in relation to survival in patients with preoperative RT

Finally, we examined the association between body composition and survival in patients with stage II and III disease who underwent preoperative RT for rectal cancer. Of these, 67 patients (74%) received short-course RT (25 Gy), 22 (24%) received long-course chemoradiotherapy (CRT; 44–50.4 Gy) and 2 (2%) received long-course RT (50 Gy). In the whole RT group, patients with low SMI had worse CSS (HR, 4.21; 95% CI, 1.32–13.36; *P* = 0.015; Fig. S1a) and OS (HR, 5.19; 95% CI, 1.87–14.40; *P* = 0.002; Fig. S1b) compared to patients with high SMI. Further analysis showed that, in patients with low SMI, short-course RT was related to better OS compared to long-course RT/CRT (HR, 0.08; 95% CI, 0.01–0.93; *P* = 0.044), although there was no difference in CSS (HR, 0.30; 95% CI, 0.07–1.23; *P* = 0.095). In patients with high SMI, there was no difference in CSS (HR, 1.58; 95% CI, 0.22–11.26; *P* = 0.645) or OS (HR, 1.55; 95% CI, 0.31–7.68; *P* = 0.592) between those undergoing short-course RT and those undergoing long-course RT/CRT.

In men with preoperative RT (*n* = 51), low SMI was related to worse CSS (HR, 5.27; 95% CI, 1.16–23.95; *P* = 0.031) and OS (HR, 7.21; 95% CI, 1.78–29.27; *P* = 0.006) compared to high SMI but not in women (*n* = 40), (CSS: HR, 3.18; 95% CI, 0.52–19.29; *P* = 0.209; OS: HR, 3.55; 95% CI, 0.79–16.06; *P* = 0.100). No significant association was observed between VAT and CSS, or OS was observed in the whole RT group or in the RT subgroup analyses by sex (Additional file 4: Table S3).

## Discussion

In this retrospective cohort study, we investigated the clinical significance of CT-measured body composition in patients with rectal cancer. Low SMI was significantly related to worse CSS and OS in the whole cohort of patients, independently of the potential confounders. Among men, low SMI was associated with both worse CSS and OS, while this association was significant only for OS among women. Low SMI was related to poor prognosis even in the preoperative RT group. Men with high VAT tended to have better CSS and OS compared to men with low VAT, and this association was significant in the group with cancer ≤ 10 cm from the anal verge. In contrast, among women, high VAT tended to be related to worse CSS than low VAT. The relationship between high VAT and decreased survival was significant in women with cancer ≤ 10 cm from the anal verge.

In line with our findings, it has been shown that sarcopenia, defined as low SMI, had a negative impact on survival in locally advanced rectal cancer [8, 9, 16]. Other studies in patients with colorectal cancer showed a similar association [4, 27–29]. A recent meta-analysis suggested that sarcopenia diagnosed with L3 SMI could be a predictor of worse survival in colorectal cancer patients [30]. The CT-based assessment of SMI appears to be a useful tool in clinical decision-making and cancer treatment planning for rectal cancer patients. The negative impact of low SMI on survival could be due to elevated systemic inflammatory response that has been associated with a poor prognosis [4, 31, 32].

Concerning the impact of visceral adiposity on clinical outcomes, our data demonstrated a lower risk of mortality among men with high VAT than among men with low VAT. On the other hand, high VAT was found to be a predictor of unfavourable survival among women. Previous studies reported no significant difference in survival rates between obese and non-obese patients with rectal cancer; however, no analyses stratified by sex were included [8, 11]. Van Baar et al. [14] observed no association between VAT and mortality in either men or women; however, they did not separate colon from rectal cancer patients in their analyses. Another study showed that visceral obesity was related to worse survival in patients with stage II colorectal cancer but better survival in stage III [33]. A study in oesophageal cancer, where 87% of the study cohort were men, showed a significant association between visceral obesity and improved survival [34]. Our present study shows a different impact of visceral obesity on survival between men and women with rectal cancer. Interestingly, it has been shown that visceral obesity was associated with lower levels of testosterone in men [35, 36], while high testosterone levels were positively associated with visceral fat accumulation in women [36]. Higher levels of testosterone were associated with an increased risk of death after cancer in men and women [37], which makes us hypothesize that the different impact of VAT on survival in men and women might be due to the testosterone-related differences in viscerally obese patients.

As preoperative RT is an established treatment against rectal cancer, we investigated the role of body composition in the subgroup of patients who underwent preoperative RT/CRT and found that low SMI predicted worse survival. Previous studies in patients receiving CRT have shown similar results [8, 9, 16], suggesting that this patient group may benefit from an intensified follow-up plan. Interestingly, in our study, among patients with low SMI, those who received short-course RT had better survival rates compared to those undergoing long-course RT/CRT. This result should be interpreted with caution, since the number of patients was small. However, these results might indicate that short-course RT may be more appropriate than long-course RT/CRT in patients with low SMI and needs further exploration.

Moreover, we found that low SMI was related to worse prognosis in stages II and III but not in stage I or IV. Since the patient numbers in each stage were limited, we need to confirm these results, especially for stages I and IV, before drawing definitive conclusions. Furthermore, in our study, men with high VAT had better outcomes compared to those with low VAT in stage IV. A previous study in patients with metastatic colorectal cancer showed that low VAT was a negative predictive marker for cancer treatment [38].

Furthermore, we found that men who were using beta-blockers at the time of diagnosis were less likely to have low SMI compared to men who were not using beta-blockers. Previous studies reported a significant association between low SMI and systemic inflammation in patients with colorectal cancer [4, 31, 32]. Sarcopenia was associated with increased levels of inflammatory cytokine interleukin-6 [39, 40], while beta-blockers were found to decrease the levels of serum interleukin-6 [19]. Taken together, our findings make us hypothesize that beta-blockers may impede skeletal muscle loss by modulating the inflammatory response usually present in cancer patients. In line with our hypothesis, treatment with beta-blockers preserved the lean body mass in a rat model [41].

Our study has several strengths. Unlike other studies that used cutoff values for the CT body composition parameters based on their own study population [16, 27], we used cutoff values specified as reference values in previous research, taking into account both sex and ethnic differences [21, 22]. This could increase the comparability of results in the field of body composition research, facilitating implementation in clinical practice. We stratified our analyses by tumour location, since upper rectal cancer is found to be related to decreased recurrence rates compared to more distal rectal cancer [15]. The impact of CT-measured body composition on clinical outcomes in rectal cancer patients has been investigated previously, but few have included the tumour location in their analyses [16, 17].

The limitations of this study need to be considered. Given its retrospective nature, we were not able to control for all potential confounders that might influence the observed associations. Moreover, our study provides evidence for the relationships we investigated, but it cannot prove causality. Only patients with an available CT colonography were included in the study, and some subgroups had a relatively small number of patients. Regarding beta-blockers, we measured their use at a one-time-point without information about their type, dose or treatment duration.

## Conclusions

In summary, the current study showed that low skeletal muscle mass, as measured by CT at the time of diagnosis, was related to a poor prognosis among both men and women with rectal cancer, even among patients who received preoperative RT. Visceral obesity had a protective effect in men with rectal cancer, especially among those with low and middle rectal cancer. In contrast, a negative association between visceral obesity and survival rates was observed in women. Our results suggest that CT-measured body composition may provide useful information for evaluating the prognosis of rectal cancer patients, while emphasizing the need to conduct the analyses according to sex and tumour location. Further evaluation in retrospective and even prospective studies with larger study populations is required to confirm our results.

## Supplementary Information

**Additional file 1: Fig. S1.** Cancer-specific survival (a) and overall survival (b) in relation to SMI in patients undergoing preoperative radiotherapy (RT).

**Additional file 2: Table S1.** Cancer-specific survival and overall survival for skeletal muscle index by stage.

**Additional file 3: Table S2.** Cancer-specific survival and overall survival for visceral adipose tissue area by stage in all patients, men and women.

**Additional file 4: Table S3.** Cancer-specific survival and overall survival for visceral adipose tissue area in patients with radiotherapy.

## Data Availability

The datasets used and/or analysed during the current study are available from the corresponding author upon reasonable request.

## Abbreviations

aHR: Adjusted hazard ratio
BMI: Body mass index
CI: Confidence interval
CRT: Chemoradiotherapy
CSS: Cancer-specific survival
CT: Computed tomography
HR: Hazard ratio
HU: Hounsfield unit
OS: Overall survival
RT: Radiotherapy
SMI: Skeletal muscle index
VAT: Visceral adipose tissue area
