# Supplementary figures and images for: Computed tomography-measured body composition and survival in rectal cancer patients: a Swedish cohort study
Source: Cancer Metab. 2022 Nov 23;10:19. doi: 10.1186/s40170-022-00297-6 (PMC9686115; doi:10.1186/s40170-022-00297-6)

## Slide 1
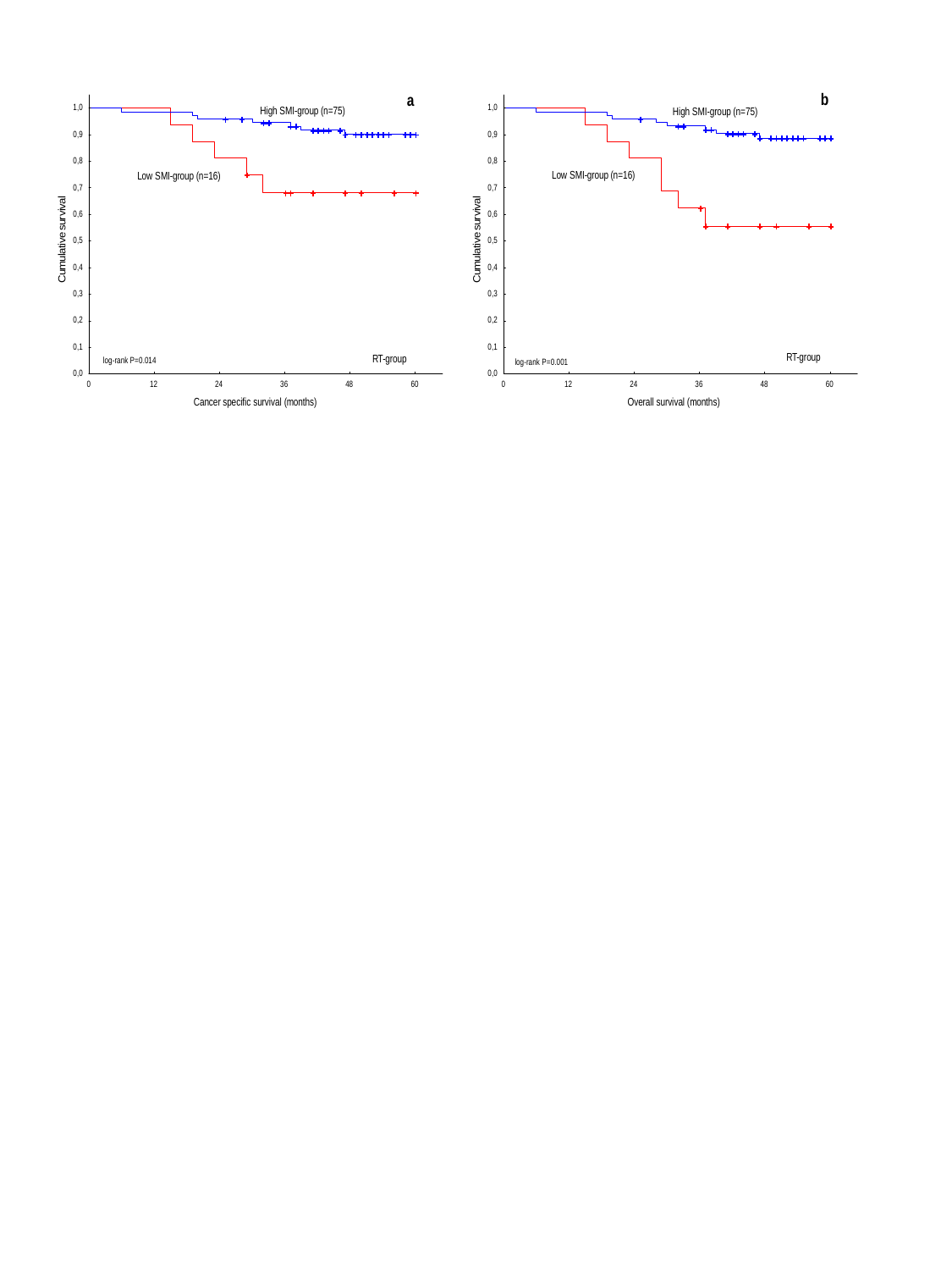

Supplement: Supplementary file 1 — Additional file 1: Fig. S1. Cancer-specific survival (a) and overall survival (b) in relation to SMI in patients undergoing preoperative radiotherapy (RT). [file 40170_2022_297_MOESM1_ESM.pptx]
